# Tissue dissociation for single-cell and single-nuclei RNA sequencing for low amounts of input material

**DOI:** 10.1186/s12983-022-00472-x

**Published:** 2022-11-12

**Authors:** Gordon Wiegleb, Susanne Reinhardt, Andreas Dahl, Nico Posnien

**Affiliations:** 1 Department of Developmental Biology, University of Goettingen, Justus-von-Liebig-Weg 11, 37077 Goettingen, Germany; 2 International Max Planck Research School for Genome Science, Am Fassberg 11, 37077 Goettingen, Germany; 3 Center for Molecular and Cellular Bioengineering (CMCB), DRESDEN-concept Genome Center, TU Dresden, Fetscherstraße 105, 01307 Dresden, Germany; 4 Goettingen Center for Molecular Biosciences (GZMB), University of Goettingen, Justus-von-Liebig-Weg 11, 37077 Goettingen, Germany

**Keywords:** Single-cell RNAseq, Single-nuclei RNAseq, *Drosophila melanogaster*, Eye-antennal disc

## Abstract

**Background:**

Recent technological advances opened the opportunity to simultaneously study gene expression for thousands of individual cells on a genome-wide scale. The experimental accessibility of such single-cell RNA sequencing (scRNAseq) approaches allowed gaining insights into the cell type composition of heterogeneous tissue samples of animal model systems and emerging models alike. A major prerequisite for a successful application of the method is the dissociation of complex tissues into individual cells, which often requires large amounts of input material and harsh mechanical, chemical and temperature conditions. However, the availability of tissue material may be limited for small animals, specific organs, certain developmental stages or if samples need to be acquired from collected specimens. Therefore, we evaluated different dissociation protocols to obtain single cells from small tissue samples of *Drosophila melanogaster* eye-antennal imaginal discs.

**Results:**

We show that a combination of mechanical and chemical dissociation resulted in sufficient high-quality cells. As an alternative, we tested protocols for the isolation of single nuclei, which turned out to be highly efficient for fresh and frozen tissue samples. Eventually, we performed scRNAseq and single-nuclei RNA sequencing (snRNAseq) to show that the best protocols for both methods successfully identified relevant cell types. At the same time, snRNAseq resulted in less artificial gene expression that is caused by rather harsh dissociation conditions needed to obtain single cells for scRNAseq. A direct comparison of scRNAseq and snRNAseq data revealed that both datasets share biologically relevant genes among the most variable genes, and we showed differences in the relative contribution of the two approaches to identified cell types.

**Conclusion:**

We present two dissociation protocols that allow isolating single cells and single nuclei, respectively, from low input material. Both protocols resulted in extraction of high-quality RNA for subsequent scRNAseq or snRNAseq applications. If tissue availability is limited, we recommend the snRNAseq procedure of fresh or frozen tissue samples as it is perfectly suited to obtain thorough insights into cellular diversity of complex tissue.

**Supplementary Information:**

The online version contains supplementary material available at 10.1186/s12983-022-00472-x.

## Background

Gene expression is a central molecular process that coordinates various aspects of organismal life, such as behavior [1] and development [2, 3]. Since differences in gene expression are often associated with variation in organismal phenotypes, comparative gene expression studies are powerful approaches to establish testable biological hypotheses [4]. For instance, differences in the expression of the developmental transcription factor genes *pitx1* and *shavenbaby* cause natural variation in armor plate formation in stickleback fish [5] and trichome formation in *Drosophila* [6], respectively. Similarly, natural variation in paternal care behavior in *Peromyscus* mice and density related stress behavior in zebrafish are tightly linked to differences in the expression of genes coding for the hormone vasopressin [7] and the neuropeptide Parathyroid hormone 2 (Pth2) [8], respectively. Advances in sequencing technologies have been facilitating extensive insights into the regulation of gene expression on a genome wide scale [9, 10]. A common observation of such studies is that gene expression strongly depends on the biological context. Spatial and temporal gene expression, for example, is tightly regulated throughout development resulting in tissue- and even cell type specific expression profiles [11–14]. In the light of this context-dependent gene regulation, it is becoming increasingly relevant to study gene expression on a cellular level.

Nowadays, multiple sequencing technologies are available allowing to quantitatively analyze the messenger RNA content of single cells [15]. Single-cell RNA sequencing (scRNAseq) has been proven powerful to reveal the cell type composition of complex tissues or organs in model organisms, such as the fruit fly *Drosophila melanogaster* [16, 17], the nematode *Caenorhabditis elegans* [18] and mouse [19]. Also, biological processes, such as development of the optic lobe of the fly brain [20], cell–cell communication in tumors [21] and immunity [22, 23] have been successfully studied. Since the analysis of scRNAseq data does not require prior knowledge of the tissue of interest, this method is exceptionally well-suited to study the cell type composition of emerging model organisms, such as sponges [24], the cnidaria *Nematostella vectensis* [25], *Hydra vulgaris* [26] and *Clytia hemisphaerica* [27], the annelid *Platynereis dumerilii* [28] and the planarian *Schmidtea mediterranea* [29, 30], the ant *Harpegnathos saltator* [31] and multiple vertebrates [32, 33]. Comparative studies have been performed to reveal divergent and conserved aspects of the motor cortex in human, marmoset, and mouse [34] and during early embryonic development in pigs, humans and cynomolgus monkeys [35].

scRNAseq protocols are composed of the following key steps [36, 37]: The tissue of interest is dissociated, and individual cells are captured either in microwell plates [38] or in micro-droplets [39]. Individual captured cells are lysed in the microwell or droplet and the released polyadenylated RNA (mRNA) is captured using poly-T oligos. The mRNA is reverse transcribed into complementary DNA (cDNA) and cell and molecule specific barcodes are added. Subsequently, sequencing libraries are generated by fragmentation, Illumina sequencing adapter ligation and amplification. The amplified libraries are eventually sequenced using next generation sequencing technologies (e.g. Illumina).

While current scRNAseq technologies allow sequencing up to 10,000 cells in one run [40], many more cells are needed as input material. For instance, mechanical stress during dissociation of complex tissue leads to increased cell death [41]. Additionally, harsh dissociation conditions using enzymes, such as Trypsin, contribute to cell damage [42, 43], altered gene expression [43, 44] and RNA degradation [45]. Due to the high cell loss during dissociation current scRNAseq methods are limited if small tissue samples are analyzed because tissue from multiple animals must be collected to obtain sufficient starting material.

**Fig. 1:**
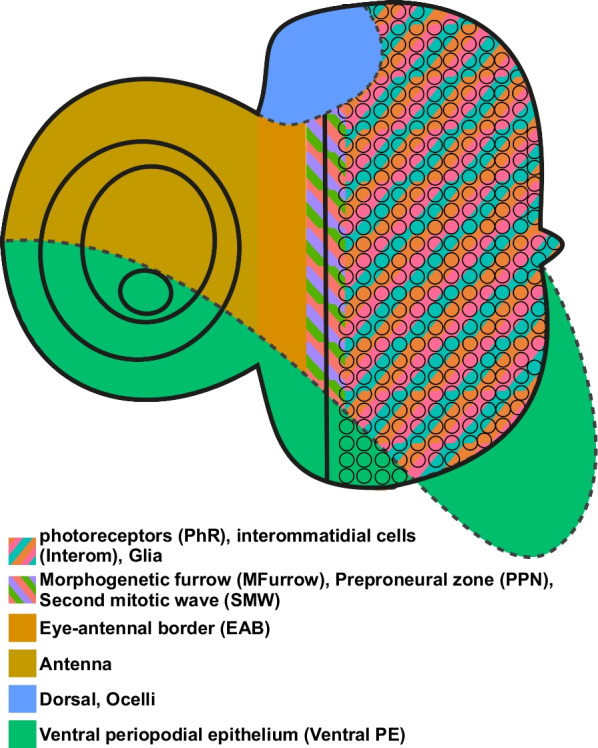
Schematic overview of major cell types of the late third instar *D. melanogaster* eye-antennal disc

Larval imaginal discs of the fruit fly *Drosophila melanogaster* are such tiny tissues. These flat epithelial sac-like tissues are specified as about 20 embryonic cells and they grow extensively during larval development to up to 44,000–60,000 cells [46–49]. During pupae stages, imaginal discs evert and give rise to external adult organs, such as wings, walking legs, genitals and compound eyes [50]. Imaginal discs are excellent models to study fundamental developmental and cellular processes, such as cell proliferation, tissue patterning and morphogenesis [51, 52]. Due to its highly heterogeneous cell type composition, the eye-antennal disc that gives rise to the compound eye, the dorsal ocelli, the antennae, and most of the head capsule [53, 54] (Fig. 1) is especially interesting for scRNAseq applications. Moreover, recent comparative work on the evolution of compound eye size and head morphology in *Drosophila* species revealed pervasive variation in these adult traits [55–62]. Accordingly, inter- and intraspecific comparisons of eye-antennal disc development have been successful in revealing underlying developmental and molecular mechanisms [59, 60, 63–65]. While gene expression in late eye-antennal disc have been studied at single cell resolution [66, 67], earlier stages are less accessible due to low cell numbers. Therefore, we evaluated different dissociation, tissue preservation and sequencing methods to establish an efficient protocol for single-cell transcriptomics in eye-antennal discs.

We show that a combination of mechanical and chemical dissociation works best to obtain sufficient and representative cells for single-cell RNA sequencing (scRNAseq). However, we observed artificial expression of stress related genes, which was most likely due to rather harsh dissociation and cell-sorting conditions. As an alternative, we tested different protocols to isolate single nuclei from fresh and frozen tissue and we show that single-nuclei RNA sequencing (snRNAseq) successfully allowed identifying key cell types without the drawback of stress-response. We find differences in the relative contribution of scRNAseq and snRNAseq to common cell types and we discuss the advantages and disadvantages of both methods. Our work provides an excellent overview of different single cell sequencing approaches when accessibility to tissue samples is limited.

## Results and discussion

### Tissue dissociation for scRNAseq with low amount of input material

For RNA sequencing of single cells (scRNAseq), heterogenous tissue samples need to be dissociated into live and intact cells. Since about 10,000 cells can be analyzed using the 10 × Genomics Chromium System and about 50% of input cells are lost throughout the preparatory steps, we first tested different dissociation protocols to obtain about 20,000 cells from entire larval organs or about 30 late third instar eye-antennal imaginal discs.

The success of different tissue dissociation protocols was evaluated by estimating the ratio of dead and live cells, as well as the final number of live cells. A dead cell staining with Trypan blue is well-established in homogeneous cell suspensions obtained from cell culture [68, 69]. However, we experienced unreliable dead/live cell ratios with our complex cell suspensions, which was most likely due to Trypan blue positive debris. Therefore, we applied a live-dead assay based on propidium iodide (PI) and Calcein green/violet to identify dead and live cells, respectively. This method allows enrichment of live cells via fluorescence activated cell sorting (FACS), which efficiently also removed debris (Additional file 1: Fig. S1A). Note that the combination of PI and Calcein violet resulted in the most efficient separation of live and dead cells due to a lower spectral overlap of both dyes during FACS. Sorted cells were examined by fluorescent microscopy to confirm that they were mostly Calcein positive and PI negative.

First, we tested purely enzymatic or mechanical dissociation protocols, respectively. Incubation of eye-antennal discs in 10 × TrypLE and 2.5 mg/ml Collagenase even for 2 h did not result in single cell solutions based on visual assessment. Imaginal discs ground with a Dounce homogenizer showed a high proportion of debris and what appeared to be single-nuclei suspensions. Additionally, different attempts resulted in inconsistent dissociation because the low amount of input tissue was barely visible and due to the manual component, it was difficult to balance complete dissociation with the destruction of cells. Based on these observations we reasoned that efficient tissue dissociation required a combination of enzymatic dissociation with gentle mechanical force.

The basic protocol was based on treatment of the tissue with TrypLE and Collagenase on a shaker at 300 rpm with pipet strokes (1000 µl pipet tips) during and after the incubation. We varied the following parameters (see Additional file 1: Table S1): enzyme concentration (1 × and 10 × TrypLE; 2.5 mg/ml and 10 mg/ml Collagenase), incubation time (10–60 min), incubation temperature (37 °C and 30 °C), number of pipet strokes (5 strokes during the incubation and 17–20 strokes after the incubation) and filtration of the cell suspension (no filter, 20 µm and 35 µm filters). 1 × TrypLE was insufficient to achieve complete dissociation in a timely manner and the addition of 10 mg/ml Collagenase resulted in an increased yield, as well as fewer cell aggregates (visual assessment). Incubation for up to 60 min at 30 °C resulted in comparable or slightly more live cells compared to a digestion at 37 °C. Filtration with a filter of 35 µm mesh size did not drastically reduce the proportion of live cells but decreased the amount of debris. The number of pipet strokes after incubation had the highest impact on cell survival with significantly reduced cell survival after more than 17 strokes. We obtained the best results with 16,208 live cells (58% survival rate) from 28 eye-antennal discs after 60 min incubation at 30 °C in 10 × TrypLE and 10 mg/ml Collagenase and 5 pipet strokes during and 17 pipet strokes after the incubation (Additional file 1: Fig. S1B). RNA extracted from this sample was of high quality (Additional file 1: Fig. S2) and suitable for 10X Genomics scRNAseq.

In summary, for low amount of input material, such as < 50 late L3 eye-antennal discs we propose a protocol that combines enzymatic dissociation in conjunction with slight mechanical disruption.

### scRNAseq reveals relevant cells and a major impact of heat shock and ribosomal genes

**Fig. 2:**
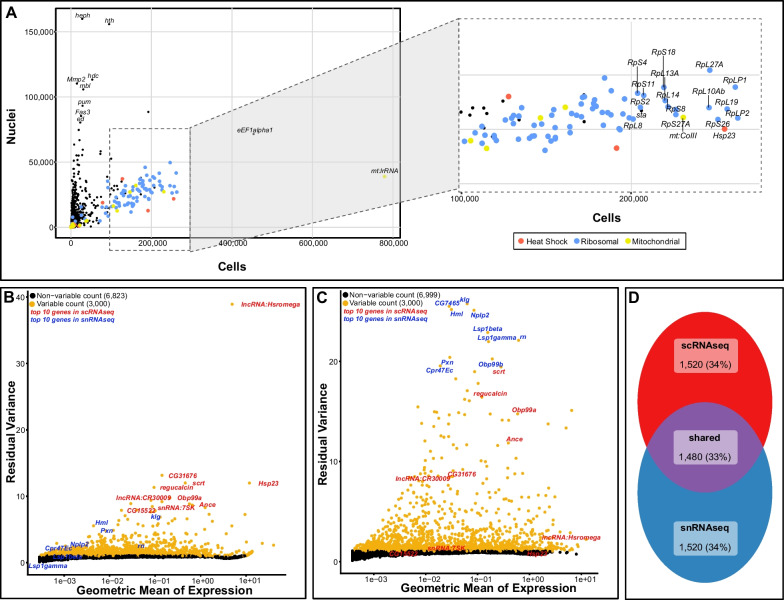
Comparison of global gene expression in scRNAseq and snRNAseq data. **A** Number of reads found in nuclei (Y-axis) over the number of reads of those same genes found in cells (Y-axis) based on normalized datasets. Ribosomal genes are highlighted in blue, mitochondrial genes are shown in yellow and heat shock genes are shown in red. Ribosomal genes are defined as genes starting RpS- or RpL- and heat shock genes are defined as genes encoding heat shock proteins, starting with Hsp-. **B**, **C** Variable feature plot of scRNAseq **B** and snRNAseq data **C** of dissociated cells and nuclei, respectively. The top 10 variable genes of the scRNAseq dataset are labeled in red and the top 10 variable genes of the snRNAseq dataset are labeled in blue. These genes are the ones having the strongest influence on clustering and cell type identification for the respective dataset (see also Fig. 3). The top 3000 genes (yellow dots) are used for subsequent cluster analyses. **D** Venn-diagram of the top 3000 variable genes in scRNAseq, snRNAseq and the overlap of both datasets. See also Additional file 9: Table S9 for a full list of genes in each category

We subjected cells obtained after FACS to a 10X Genomics Chromium run to test if the established dissociation protocol resulted in representative cell types expected in the eye-antennal disc. After droplet-based isolation of RNA from individual cells and subsequent Illumina sequencing, we obtained almost 200 million reads from about 14,500 cells with 13,303 reads and 537 genes per cell (Table 1). 12,000 cells showed less than 10% mitochondrial gene expression (Additional file 1: Fig. S3A) confirming that we mostly isolated live cells. Many reads of the scRNAseq dataset mapped to genes coding for heat shock proteins (Fig. 2A) and among the top ten genes with most variable expression across cells, we found two heat shock related genes (*Hsp23* and *lncRNA:Hsromega*, Fig. 2B). To evaluate whether our rather high dissociation temperature of 30 °C may impose stress on the cells, we re-evaluated a previously published scRNAseq dataset for eye-antennal discs that is based on dissociation at room temperature [66] (personal communication). As the list of genes expressed in different cell types contains 19 heat shock related genes of which three (*Hsp27, Hsp26 and Hsp83*) show moderate to high expression in almost all cell types [66 Supplementary Data 2], we suggest that other steps of the dissociation protocol may be responsible for the observed stress response. The distribution of reads also showed a high expression of cytoplasmic genes, such as *eEF1alpha1* and eukaryotic elongation factors (Fig. 2A). Additionally, a lot of genes coding for ribosomal proteins were expressed in our dataset (Fig. 2A). The high content of ribosomal genes is expected for scRNAseq because cytoplasmic mRNA is extracted and ribosomal mRNAs are known to be very stable [70, 71]. However, they are often considered uninformative.

**Table 1:** Summary statistics for the cell- and nuclei dataset

Dataset	scRNAseq	snRNAseq
Estimated number of cells (# of discs)	14,487 (28)	9048 (41)
Number of cells per disc (% of expected number of cells^a^)	517 (1.1)	221 (0.5)
Median genes per cell	537	812
Mean reads per cell	13,303	13,334
Valid barcodes	96.10%	97.10%
Number of reads	192,731,871	120,649,741
Fraction of reads in cells	38.20%	73.50%
Total genes detected	11,062	12,296
Median UMI counts per cell	1249	1383
Reads mapped to genome	93.20%	85.70%
Reads mapped confidently to genome	86.40%	84.50%
Reads mapped confidently to intronic regions	2.10%	14.70%
Reads mapped confidently to intergenic regions	9.00%	0.80%
Reads mapped confidently to exonic regions	75.20%	69.00%
Percentage of cells with high mitochondrial read count (> 10%)	14.00%	0.02%

^a^Assuming that a late third instar eye-antennal disc is composed of about 44,000 cells[49]

We performed an unbiased cluster analysis based on variable gene expression and performed dimension reduction using Uniform Manifold Approximation and Projection (UMAP) [72]. We obtained 22 cell clusters which we annotated based on marker genes used in previous scRNAseq analyses [66, 67] and based on prior knowledge from the literature (see Materials and Methods for details, Additional files 2, 3, and 4: Tables S2 and S3 and Fig. S4). Gene ontology (GO) term enrichment analyses for marker genes defining each of these 22 clusters (Additional file 4: Table S4) suggest that cells in each cluster expressed genes involved in relevant biological processes (Additional file 5: Table S5).

**Fig. 3:**
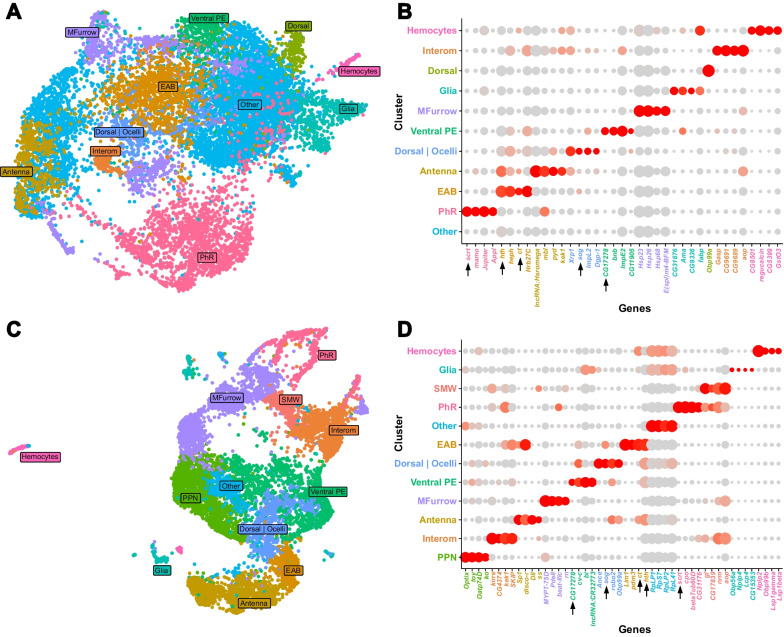
Most variable genes and marker genes for cell clusters in scRNAseq and snRNAseq. **A** UMAP of scRNAseq data. The cluster annotations are based on a combination of similar clusters to increase comparability between datasets. See Additional files 1 and 3: Fig. S4 and Table S3 for the annotation of all clusters prior to cluster merging. **B** Dot plot of the top four marker genes (X-axis) for each cell cluster (Y-axis) obtained after scRNAseq. The size of the dots represents the percentage of cells expressing each gene. The color intensity represents the average expression level. **C** UMAP of snRNAseq data. The cluster annotations are based on a combination of similar clusters to increase comparability between datasets. See Additional files 1 and 3: Fig. S7 and Table S3 for the annotation of all clusters prior to cluster merging. **D** Dot plot of the top four marker genes (X-axis) for each cell cluster (Y-axis) obtained after snRNAseq. The size of the dots represents the percentage of cells expressing each gene. The color intensity represents the average expression level. The gene names on the dot plots are color coded by the cluster they are enriched in, and arrows point to genes that are shared in similar clusters in scRNAseq **B** and snRNAseq **D** data. Note that the color code of the cluster identities is consistent among figures in the main text

**Fig. 4:**
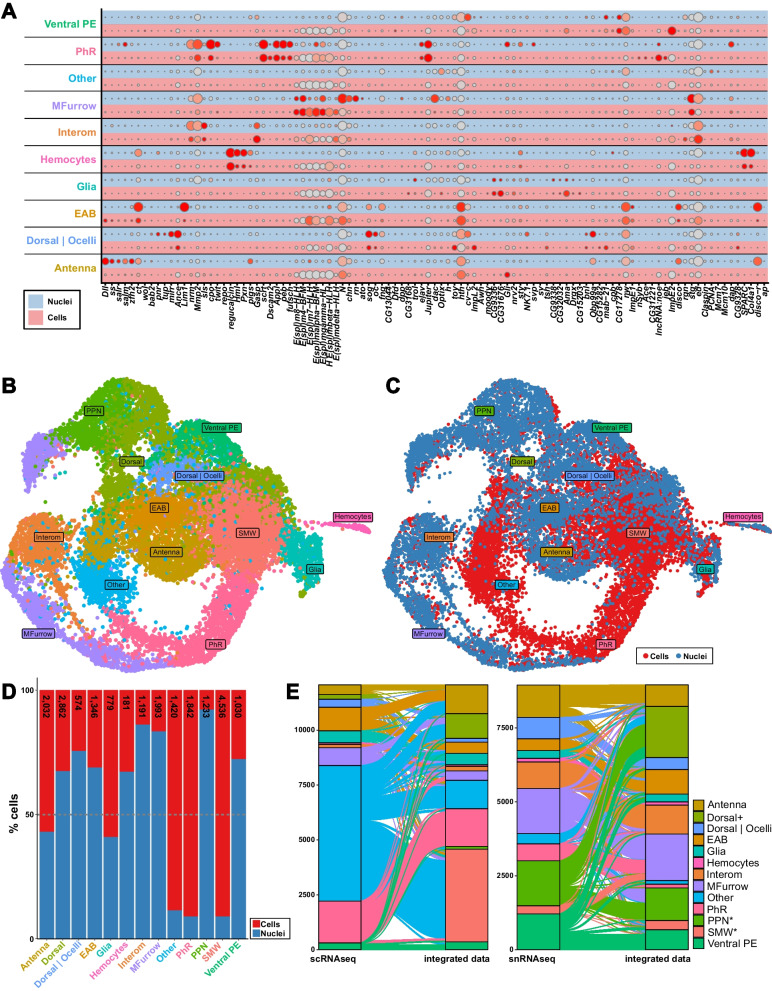
Direct comparison of scRNAseq and snRNAseq datasets on the level of major cell types. **A** Dot plot showing the expression of all genes used as cell type markers (Additional file 2: Table S2) in scRNA (red lanes) and snRNA data (blue lanes) split by cell type. The color intensity of the dots represents the expression level of a gene in a cluster and the size of the dots represent the percentage of cells in a cluster expressing the respective gene. **B**, **C** UMAPs of integrated scRNAseq and snRNAseq data. Cells are colored by cluster identity **B** or the initial dataset (i.e. scRNAseq and snRNAseq) **C**. The cluster annotations are based on a combination of similar clusters. See Additional files 1 and 3: Fig. S9 and Table S3 for the annotation of all clusters prior to cluster merging. **D** Proportion of cell or nuclei contribution to each cluster by dataset. The total number of cells in each cluster is provided for each cell type at the top of the bar plot. **E** Alluvial plots depicting changes of cell identities between separately analyzed scRNAseq (left) and snRNAseq (right) data and integrated data. The Y-axes indicate the number of cells (left) and nuclei (right) of the separately analyzed datasets

For the sake of comparability (see below), we manually combined similar cell types (see Additional file 3: Table S3) to obtain 11 clusters (Fig. 3A) representing most cell types that have been previously described in scRNAseq data for eye-antennal discs [66, 67] (Fig. 1). To validate our automatic cluster annotation, we tested whether relevant genes were among the top four genes that define a certain cluster. For instance, *cut (ct)* [73, 74] and *homothorax (hth)* [73] have been shown to be involved in antennal development and they are among the top four genes expressed in antennal and eye-antennal border (“EAB”) clusters (Fig. 3B). In line with previously reported roles in photoreceptor differentiation [75, 76], we found *amyloid protein precursor-like* (*Appl*) and *scratch* (*scrt*) among the top four genes in photoreceptors (“PhR”) (Fig. 3B). And in the morphogenetic furrow (“MFurrow”) cluster, we observed a member of the enhancer of split gene complex (*E(spl)m4-BFM*) (Fig. 3B), which are broadly expressed in this tissue [77, 78]. Besides these relevant biological findings, the potential stress response of the cells was also evident in our cluster analysis because three heat shock genes (*Hsp23*, *Hsp26* and *Hsp68*) were among the top four cluster defining genes in the “MFurrow” cluster and those three genes were expressed in most cells of all clusters (Fig. 3B). Note that the “Other” cluster was composed of diverse cell types, such as antennal, dorsal and second mitotic wave (“SMW”) cells (see Fig. 4E, left panel) and accordingly, this cluster was not clearly defined by a set of marker genes (Fig. 3B).

In summary, our tissue dissociation protocol successfully resulted in a cell suspension containing major cell types of the eye-antennal disc but may pose stress on the cells which is detectible through high expression of heat shock genes. Additionally, the high level of ribosomal genes may introduce a bias during further analysis of such data.

### Cryo-preservation of imaginal discs for efficient isolation of single nuclei

Our scRNAseq data suggested that the applied dissociation conditions were still stressful for the cells. Additionally, the protocol relies on the processing of fresh tissue samples hampering the analysis of even smaller tissue samples. For instance, eye-antennal discs at the late L3 larval stage contain 44,000–60,000 cells, while discs at the transition from the L2 to the L3 stage are only composed of about 5000 cells [79]. Therefore, about 12 times more discs are needed to obtain sufficiently high cell numbers for scRNAseq applying our single cell dissociation protocol. As tissue growth is an integral part of developmental processes, more efficient protocols are needed to harness the full potential of single cell sequencing methods for developmental biology. To this end, we tested two main approaches: First, we evaluated the use of single nuclei for RNA sequencing (i.e. snRNAseq) as snRNAseq has been shown to result in comparable data, especially for tissue samples that are difficult to dissociate into single cells [80–83]. Second, we tested the effect of cryo-preservation on the subsequent isolation of single nuclei and RNA integrity as this step allows collecting small tissue samples over time.

For the isolation of single nuclei, we tested two main protocols: One protocol suggested by 10X Genomics is based on NP40 as detergent and a small number of centrifugation and pipetting steps [84]. The other protocol had been established for human heart tissue and is based on using Triton X-100 as a detergent and a variety of RNAse inhibitors to preserve RNA in single nuclei [85]. When 30–50 freshly dissected eye-antennal discs at late L3 stage were used for nuclei isolation, both protocols resulted in more than 20,000 nuclei and extracted RNA was of high quality suitable for snRNAseq (Additional file 1: Fig. S5).

We next dissected imaginal discs, snap-froze them in liquid nitrogen and stored them at − 80 °C for at least one day, or up to four weeks. All applied protocols allowed us to isolate more than 20,000 intact nuclei from about 30 cryo-preserved eye-antennal discs. RNA extracted from nuclei isolated with the 10X Genomics protocol resulted in low RNA quality, suggesting a high level of RNA degradation (Additional file 1: Fig. S6, lanes 7 and 8). The addition of Citric acid to the dissociation buffer has been shown to preserve RNA integrity in human pancreatic cells [86]. However, the use of Citric acid in the 10X Genomics protocol did only marginally improve the quality of RNA extracted from cryo-preseved samples (Additional file 1: Fig. S6, lanes 10 and 11). In contrast, we observed almost no RNA degradation and high RNA quality when we used the protocol that employs RNAse inhibitors (Additional file 1: Fig. S6, lanes 1, 2, 4 and 5). RNA integrity was preserved even when eye-antennal discs were thawed for 3.5 h and frozen again prior to nuclei isolation and RNA extraction (Additional file 1: Fig. S5), showing that the use of RNAse inhibitors are highly efficient to prevent RNA degradation when processing of cryo-preserved tissue samples. Based on the high yield and the high RNA quality, we conclude that the combination of cryo-preservation and nuclei isolation employing RNAse inhibitors is highly efficient to process low input material for snRNAseq.

### snRNAseq identifies eye-antennal disc gene expression and reduces technical biases

To test if the nuclei obtained after cryo-preservation are suitable for snRNAseq and represent main cell types of the eye-antennal disc, we subjected the nuclei to a 10X Genomics run to obtain 120 million reads from about 9000 cells with 13,334 reads and 812 genes per cell (Table 1). For the analysis of the data, we applied the same pipeline and settings as for the scRNAseq dataset, with the exception that intronic reads were included because pre-mRNA is expected in nuclei [83]. Among the 10 genes with most variable expression in the dataset (Fig. 2C), we found some with known functions and expression in late L3 eye-antennal discs. For instance, the homophilic cell adhesion molecule Klingon (Klg) is strongly expressed during R7 photoreceptor development [87] and *rotund* (*rn*) that codes for a Kruppel zinc-finger transcription factor is expressed in large parts of the antennal field [88]. Importantly, we did not observe genes associated with heat shock response among the top 10 variable genes (Fig. 2C) and no bias of reads originating from heat shock genes was observed (Fig. 2A), suggesting that they will not impact subsequent clustering analyses as observed for the scRNAseq data. As expected for snRNAseq data [80, 82, 89, 90], we found only two cells with more than 10% mitochondrial gene expression (Additional file 1: Fig. S3B) and 28% fewer reads originating from ribosomal genes (Fig. 2A) compared to scRNAseq, suggesting lower impact of such uninformative reads on the entire dataset.

An unbiased clustering of the snRNAseq data using the same resolution as for scRNAseq (see above) resulted in 24 unique clusters, which we annotated using the same pipeline as described for the scRNAseq data (Additional file 1: Fig. S7). GO term enrichment analyses for marker genes defining each of these 24 cell clusters (Additional file 6: Table S6) revealed biological processes relevant for eye-antennal disc cells (Additional file 7: Table S7).

To improve comparability of the snRNAseq and scRNAseq data (see below), we combined similar clusters (see above & Additional file 3: Table S3), and we obtained the same clusters as for scRNAseq data (Fig. 3B). Additionally, we found clearly assigned clusters for second mitotic wave (“SMW”) and preproneural (“PPN”) cells, which were missing from the scRNAseq data (compare Fig. 3A to C). A closer examination of the top four genes that define a certain cluster revealed for instance *Sp1* [91], *disco-related* (*disco-r*) [92], *Distalless* (*Dll*) [93] and *spineless* (*ss*) [94] in the antennal cell cluster (Fig. 3D) and indeed all four genes have been implicated in antennal development. In line with their role in retinal differentiation and their expression in retinal progenitors we found *optix/six3* [95] and *twin of eyeless* (*toy*) [96] among the top four genes in the preproneural (“PPN”) cell cluster. *glass* (*gl*) [97–99], *scratch* (*scrt*) [76] and *anterior open* (*aop*) [100–102] have been implicated in late retinal differentiation processes and accordingly, we observed highly specific expression in cells posterior of the morphogenetic furrow (i.e. second mitotic wave, “SMW”; photoreceptors, “PhR” and interommatidial cells, “Interom”). Note that the “Other” cluster was largely defined by ribosomal gene expression (Fig. 3D). As ribosomal mRNAs are more stable in the cytoplasm, we hypothesize that all nuclei that still had remnants of cytoplasm attached show higher levels of ribosomal mRNAs compared to pure nuclei and thus lead to the distinct expression profile and clustering result.

Overall, we conclude that the snRNAseq dataset captured gene expression profiles of eye-antennal disc cells and at the same time, technical artifacts, such as heat shock gene expression and an excess of reads from ribosomal genes were diminished.

### scRNAseq and snRNAseq show largely overlapping gene expression profiles

Besides the reduced expression of mitochondrial, ribosomal and heat shock genes in the snRNAseq dataset, we wanted to compare the snRNAseq and scRNAseq datasets more thoroughly. The comparison of the top four genes defining comparable cell clusters in the scRNAseq and snRNAseq datasets revealed five shared genes (arrows in Fig. 3B, D), suggesting that cell-type specific expression is variable in our two datasets. To characterize the overlap of gene expression profiles further, we first asked to what extent the most variable genes overlapped in both datasets. Most of the top ten variable genes in the scRNAseq dataset were also among the most variable genes in the snRNAseq dataset and vice versa (Fig. 2B, C; Additional file 8: Table S8). Among the top 3000 most variable genes, 1480 were present in both datasets, while 1520 genes were unique for the scRNAseq or the snRNAseq dataset, respectively (Fig. 2D; Additional file 9: Table S9). The genes unique for scRNAseq or snRNAseq predominantly represented general cellular and metabolic processes (Additional files 1 and 9: Fig. S8A, B, Table S9), while the shared genes were highly enriched for developmental processes relevant for larval eye-antennal discs (Additional files 1 and 9: Fig. S8C, Table S9). This finding suggests that cell type specific genes may largely be shared between datasets. To confirm this, we compared two types of gene sets.

First, a direct comparison of the manually curated marker genes used for automatic cluster annotation (Additional file 2: Table S2) showed similar expression in comparable clusters between both datasets (Fig. 4A). Exceptions were the antennal marker genes *Dll, ss, salr, salm, zfh2, ct* and *disco-r*, which were highly expressed in the antennal (“Antenna”) and eye-antennal boarder (“EAB”) clusters in the snRNAseq and scRNAseq dataset, respectively (Fig. 4A). This observation suggests that these two cell types were not clearly distinguishable in the scRNAseq data.

Second, we asked for each of the comparable clusters (i.e. clusters defined in Fig. 3A and C) how many of the differentially expressed genes (FDR 0.05 and log2-fold change > 0.25) were shared between datasets. Depending on the cell type, we found 6% (e.g. “Antenna” and “Ventral PE”) to 26% (e.g. “PhR” and “Hemocytes”) overlap (Additional files 1 and 10: Fig. S9, Table S10). Among the genes that were shared between datasets, we observed factors with well-established functions in the respective cell types, such as *glass* (*gl*) [97–99], *scratch* (*scrt*) [76] and *amyloid protein precursor-like* (*Appl*) [75] in photoreceptors, *cut (ct)* [73, 74] and *homothorax (hth)* [73] in antennal and eye-antennal border cells and members of the *enhancer of split* gene complex [77, 78] in the morphogenetic furrow. Our observation is well in line with a comparison of scRNAseq and snRNAseq data in heterogeneous human liver tissue that also showed cell type specific correlation coefficients in log2-fold changes among the two datasets (coefficients ranging from 0.06 to 0.66) [103]. Therefore, despite fundamental differences in the two applied sequencing approaches, we found that global and cluster-specific gene expression was considerably comparable between our scRNAseq and snRNAseq datasets.

### scRNAseq and snRNAseq show differences cell type composition

To directly compare the snRNAseq and the scRNAseq datasets, we combined both datasets by clustering cells and nuclei based on expression profiles and performing dimension reduction. The obtained 21 clusters were annotated based on our manually curated marker gene list (Additional files 1, 2 and 3: Fig. S10, Table S2 and Table S3) and similar clusters were combined as described above for the individual datasets (Fig. 4B; Additional file 3: Table S3). Based on our cluster (i.e. cell type) annotation, we found that the combined dataset contained all major cell types that have been previously described in scRNAseq data for eye-antennal discs [66, 67] (Fig. 4B, Fig. 1).

The UMAP of the integrated data with cells color-coded by the dataset suggested that all major cell types were represented in both datasets and that the different datasets contributed unequally to some cell types (Fig. 4C). Therefore, we quantified the number of cells of each cluster originating from scRNAseq and snRNAseq data, respectively (Fig. 4D). This analysis confirmed the biases in the data composition as 8 of the 13 clusters were predominantly defined by snRNAseq data and 5 clusters were defined by scRNAseq data (Fig. 4D). Clusters with a strong scRNAseq-bias were the ones with ambiguous cell type assignment (“Other”), second mitotic wave (“SMW”) and photoreceptors (“PhR”) (Fig. 4D). The significant contribution of the scRNAseq dataset to second mitotic wave (“SMW”) cells was particularly unexpected as we were unable to unequivocally identify this cell type in the individual scRNAseq dataset (Fig. 3A). To test, which other cell type in the scRNAseq data may contribute to the “SMW” cluster in the integrated dataset, we used the cell identifiers to compare the cluster assignment of each cell in the individual scRNAseq dataset to the integrated dataset (Fig. 4E, left panel). This analysis revealed that cells defined as second mitotic wave (“SMW”) cells in the integrated dataset were part of the large “Other” cluster in the scRNAseq data. Moreover, this analysis showed that the “Other” cluster also contained preproneural (“PPN”) cells (Fig. 4E, left panel), which we could not find in the individual scRNAseq dataset (Fig. 3A). This observation suggests that the scRNAseq data did not contain sufficient information to clearly define these two cell types.

Since we were unable to identify a clear “Dorsal” cluster in our snRNAseq data, we used the specific nuclei identifiers to track individual nuclei between the snRNAseq data and the integrated data (Fig. 4E, right panel). The alluvial plot showed that “Dorsal|Ocelli”, ventral peripodial epithelium (“Ventral PE”), preproneural (“PPN”) and almost all “Other” nuclei contributed to the “Dorsal” cluster in the integrated dataset. This heterogeneous contribution is expected as we combined multiple clusters containing different cell types with dorsal identity for the comparative analysis (Additional file 3: Table S3). For instance, the peripodial epithelium covers the entire eye-antennal disc [104] and preproneural cells are present along the entire dorsal–ventral axis [105]. Accordingly, both cell types may contribute to dorsal cells.

Generally, the alluvial plots showed that some cell types could be clearly defined in the two different datasets. For instance, photoreceptors (“PhR”) and ventral peripodial epithelium (“Ventral PE”) cells of the scRNAseq dataset, eye-antennal border (“EAB”) and antennal cells of the snRNAseq dataset and interommatidial cells (“Interom”) of both datasets were almost fully recovered as such in the integrated data. For other clusters the cell type assignment in the individual datasets was less clear, which may be caused by the observed differences in cluster-specific gene expression (see above).

In summary, our direct comparison of scRNAseq and snRNAseq data showed that both methods identified major cell types present in the eye-antennal disc, but we observed differences in the relative contribution of the datasets to different cell types. While previous comparisons of scRNAseq and snRNAseq also showed such biases [80, 103, 106, 107], the observed discrepancy between our two datasets could partially be explained by experimental difference. Specifically, larvae for scRNAseq were staged based on morphology and behavior (i.e. late wandering larvae at the transition to white prepupae) and discs for snRNAseq were dissected from larvae staged by developmental time (120 h after egg laying). Therefore, we assume that the larvae used for scRNAseq had been slightly older than those obtained for snRNAseq.

## Conclusion

Assessing genome wide gene expression for individual cells has proven powerful to describe the heterogeneity of complex tissues, identify novel cell types and to study biological processes, such as immunity and cell–cell interactions at unprecedented detail. Despite the technological advances, single-cell RNA sequencing (scRNAseq) methods still require many cells as starting material. Therefore, we evaluated different dissociation protocols and compared scRNAseq to single-nuclei RNA sequencing (snRNAseq) with special emphasis on low-input material. Based on data obtained for eye-antennal imaginal discs of *Drosophila melanogaster*, we found snRNAseq superior to scRNAseq for the following reasons: (1) The isolation of nuclei requires fewer experimental steps compared to tissue dissociation into live cells, increasing reproducibility across experiments. This feature is especially relevant if gene expression comparisons are needed on the level of individual cells, for example to assess the effect of experimental manipulations, to study different developmental stages or to compare species/populations. (2) We observed significantly reduced stress-related expression responses and in line with a previous report in human liver data [103] we found reduced ribosomal and mitochondrial gene expression in snRNAseq data, suggesting that more informative reads contribute to biological insights. (3) We showed highly efficient nuclei isolation and high-quality RNA extraction from frozen tissue [see also e.g. 108]. It is a major advantage to have the opportunity to collect tissue over time and process samples simultaneously, especially for low-input material. (4) In line with previous reports [80, 82, 83, 89, 90, 107, 109, 110], our snRNAseq dataset contained sufficient expression information to unravel almost all major cell types expected in eye-antennal imaginal discs. We only lacked a clearly defined cluster of dorsal cells in our snRNAseq data, while we were unable to unequivocally identify important second mitotic wave (“SMW”) and preproneural (“PPN”) cells in our scRNAseq. (5) While scRNAseq has been shown to result in biased cell composition, due to different cell sizes, shapes, and survival rate upon dissociation [111], the more streamlined nuclei isolation procedure should ensure a more representative assessment for snRNAseq, especially for complex organs, such as nervous tissue [81]. For instance, we found indications that snRNAseq may be more efficient in capturing rather complicated cell types, such as the large polyploid cells of the peripodial epithelium (“Ventral PE” in Fig. 4D).

It is important to consider major differences in the analysis and interpretation of scRNAseq and snRNAseq data. For instance, snRNAseq data contains intronic reads originating from immature nuclear RNA [83]. Accordingly, well-annotated genome resources are advantageous and analyses pipelines need to be adjusted to also include reads mapped to introns in subsequent read quantification. snRNAseq data captures only rather transient nuclear RNA, while scRNAseq also includes cytoplasmic mature mRNA. Hence, gene regulation events acting on the level of nuclear export [112, 113], splicing [114] or mRNA maturation [115, 116] may contribute to differences in expression information derived from nuclei and cells, respectively. If cytoplasmic RNA molecules are of special interest and thus single cells need to be isolated, we strongly suggest a dissociation protocol combining chemical and mechanical treatment of tissue samples in conjunction with FACS-aided life cell selection based on fluorescent life-dead cell staining. Our direct comparison of scRNAseq and snRNAseq data showed that the different datasets contributed differently to the obtained cell types. While some of these differences can be explained by slightly different larval staging procedures in our experiments, it remains to be established, which exact cellular or molecular features influence the more efficient recovery of certain cell types in sc/snRNAseq. In the light of our findings, it will be important to stick to either scRNAseq or snRNAseq if comparative questions are tackled.

In summary, based on a thorough evaluation of different dissociation and sequencing protocols we suggest a highly efficient snRNAseq procedure to obtain high-quality expression data for individual nuclei. Our procedure is specifically tested for low-input material and will therefore be perfectly suited for future studies with limited access to tissue samples.

## Methods

### Fly stock keeping and tissue dissection

Flies of the Oregon R strain of *Drosophila melanogaster* were kept in fly food vials (400 g of malt extract, 400 g of corn flour, 50 g of soy flour, 110 g of sugar beet syrup, 51 g of agar, 90 g of yeast extract, 31.5 ml of propionic acid, and 7.5 g of Nipagin dissolved in 40 ml of Ethanol, water up to 5 L) in incubators at 25 °C prior to the experiment for at least one generation. The incubators maintain 12 h light and dark cycles. For single-cell RNA sequencing (scRNAseq), eye-antennal discs were dissected from late wandering L3 larvae. For single-nuclei RNA sequencing (snRNAseq), eye-antennal discs were dissected from late L3 larvae at 120 h after egg laying. To control for larval density, eggs were deposited on yeast-coated apple agar plates for 1–2 h and incubated for 24 h before 40 first instar larvae were transferred to food vials for further incubation.

For each dissociation experiment, at least 15 larvae were dissected for no more than 1 h in 400 µl ice-cold 1 × PBS. When larval tissue was used for tests, the larvae were everted, and a mix of inner organs (e.g. imaginal discs, gut, brain etc.) was isolated. When eye-antennal discs were used, ~ 30 eye-antennal discs were dissected (generally from about 15 larvae). All organs were transferred into a microcentrifuge tube containing Storage Buffer (4% BSA, 0.2U Protector RNAse inhibitor (Merck; 3,335,399,001) in PBS). If the sample was to be frozen for later nuclei extraction, the tube was submerged in liquid nitrogen for 2 min and stored at − 80 °C until further processing.

### Recommended dissociation protocol to obtain single-cell suspensions for scRNAseq

In the following, the dissociation protocol is described that was used to obtain the live cells used for scRNAseq. Additional file 1: Table S1 contains detailed information about the protocol steps that had been varied and tested to achieve efficient dissociation.

 About 30 eye-antennal discs were dissociated in 10 × TrypLE (Thermo Fisher Scientific; A1217701) containing 2.5 mg/ml Collagenase (Invitrogen; 17,100,017) for 30 min on the shaker at 30 °C and 300 rpm. Every 15 min, or, if the digestion time was only 15 min, once after 7.5 min, the discs were pipetted up-and-down 5 times using a 1000 µl pipet tip to dissociate cell clumps efficiently. The reaction was stopped using Schneider’s supplemented medium (SSM, 0.02 mg/ml Insulin in Schneiders Medium (Thermofisher/Gibco; 21,720-024)). The suspension was gently pipetted up and down ~ 17 × with a 1000 µl pipet tip and passed through a 35 µm cell strainer (Corning; 352,235). The suspension was centrifuged for 5 min at 1000 rcf. For low amounts of tissue, the pellet might be small and barely visible on the side wall of the tube. Therefore, it is advantageous to use a swing bucket centrifuge to ensure that the pellet accumulates in the center at the bottom of the tube. The supernatant was removed, and the pellet was resuspended in 1 × PBS. The suspension was centrifuged again (see above), the supernatant was removed, and the pellet was resuspended in 0.04% BSA (Invitrogen; 17,100,017) and 0.2 U/µl Protector RNAse Inhibitor (Sigma-Aldrich; 3,335,402,001) in 1 × PBS.

The cells were stained depending on the application: For testing non-fluorescent live-dead assays, 10 µl of a cell suspension were mixed with 10 µl Trypan Blue (Invitrogen; 15,250,061). 10 µl of this solution were transferred onto a counting chamber and cells were counted using a Zeiss Telaval 31. For fluorescence-based assays, Calcein-AM (green: Sigma-Aldrich; 56,496 or violet: Sigma-Aldrich; ThermoFisher Scientific; C34858) was used to stain live cells at a final concentration of 0.5 µg/ml. The suspension was incubated for 30 min to 1 h in the dark at room temperature on a shaker. Either DAPI or Propidium Iodide were used to stain nuclei at a final concentration of 1 μg/ml each and incubated for 10–30 min. The cell suspension was then immediately processed by Fluorescence Activated Cell Sorting (FACS) at the Universitätsmedizin Göttingen or at the Center for Molecular and Cellular Bioengineering Dresden using a Becton Dickinson BD FACSAria™ II Cell Sorter or BD FACSAria™ III Cell Sorter. In consecutive gating steps, living cells were selected out from debris, damaged cells, and doublets. Events which were positive for Calcein, as well as negative for Propidium Iodide were interpreted as live, undamaged cells. After FACS, cells were visually inspected under the microscope, counted and the volume of the suspension was adjusted with PBS and 0.04% BSA to achieve a concentration of ~ 1,000 cells per µl to match the optimal requirements for 10X Genomics scRNAseq.

### Recommended dissociation protocol to obtain single nuclei for snRNAseq

Frozen tissue was thawed at 4 °C and kept on ice for the following steps unless specified otherwise. The tissue was transferred into a precooled Dounce homogenizer (2 ml) and 500 µl of Homogenization Buffer (HB) (0.4 U/µl RiboLock RNase Inhibitor (ThermoFisher Scientific; EO0381), 0.2 U/µl SUPERase In™ RNase Inhibitor (ThermoFisher Scientific; AM2694), 0.10% (v/v) Triton X-100 in NIM2; Nuclei isolation buffer 2 (NIM2): 1 µM DTT, 1 × Protease Inhibitor (Promega; G6521) in NIM1; Nuclei isolation buffer 1 (NIM1): 250 mM Sucrose, 25 mM KCl, 5 mM MgCl_2_, 10 mM Tris HCl, ph 8 in nuclease free water) was added. The tissue was homogenized with 8 strokes of the tight pestle and kept on ice whenever possible. If the homogenization seemed incomplete after visual inspection, 1 stroke was added at a time up to a maximum of 11 strokes. The homogenized tissue was filtered through a 30 µm MACS SmartStrainer (Miltenyi; 130-098-458) to exclude larger debris. The homogenizer was furthermore washed with 2 × 500 µl of HB to transfer as much of the tissue as possible to the cell strainer. The nuclei suspension was centrifuged at 500 g for 5 min at 4 °C in a swing bucket centrifuge to obtain a nuclei pellet. The supernatant was removed, and the pellet was resuspended in 500 µl Storage Buffer.

For subsequent FACS either 5 µl of a 100 μg/ml DAPI solution (Carl Roth; 6335.1) or 1 drop of NucBlue™ (Hoechst 33,342; Invitrogen Live ReadyProbes™; R37605) was added and the nuclei were incubated for 10–20 min for the staining to occur. During the exposure time of the staining, the sample was immediately transferred to FACS (Becton Dickinson (BD™) FACS Aria III Flow Cytometry Cell Sorter) to collect intact nuclei into a 1.5 ml microcentrifuge tube pre-coated with 1% BSA containing 0.04% BSA in 5 µl PBS. The concentration should be ~ 1,000 nuclei per µl to match the optimal requirements for 10X Genomics snRNAseq. The gates were set to select for DAPI positive nuclei. Particles smaller than 1 µm were excluded to remove small debris and damaged nuclei. Doublets and irregular shaped debris were also filtered out through gating as much as possible. Nozzle size was 100 µm. FACS was performed at the Universitätsmedizin Göttingen or at the Dresden Concept Genome center using a Becton Dickinson BD FACSAria™ II Cell Sorter or BD FACSAria™ III Cell Sorter. The settings were adjusted using unstained and stained samples.

### Library preparation and 10 × genomics sequencing

scRNAseq and snRNAseq were performed at the Dresden Concept Genome Center on a 10 × Genomics Chromium sequencing system. The viability of the sorted cells or quality of nuclei were visually inspected under a light microscope (with 200 × magnification) from a small aliquot of cells or nuclei stained with Trypan blue.

Up to 20,000 cells/nuclei were carefully mixed with reverse transcription mix using the Chromium Single Cell 3’ Library & Gel beads chemistry v3 (10X Genomics, PN 1,000,075) and loaded into a Chromium Single Cell B Chip (10X Genomics, PN 1,000,073) on the 10X Genomics Chromium system [117].

Following the guidelines of the 10X Genomics user manual, the droplets were directly subjected to reverse transcription, the emulsion was broken, and cDNA was purified using Dynabeads MyOne Silane (10X Genomics). After cDNA amplification (11 cycles for cells, 12 cycles for nuclei), the sample was purified and underwent a quality control check on the Fragment Analyzer.

Preparation of single-cell or -nuclei RNA-seq libraries (fragmentation, dA-Tailing, adapter ligation and an indexing PCR step with 12 cycles (cells) or 15 cycles (nuclei)) followed the manufacture’s recommendations. After quantification, the libraries were sequenced on an Illumina NextSeq 500 using a high-output flowcell in PE mode (R1: 28 cycles; I1: 8 cycles; R2: 56 cycles) or on the Illumina Novaseq 6000 system with a S2 flowcell in PE mode (R1: 28 cycles; I1: 8 cycles; R2: 94 cycles). An average of 13,000 fragments per cell were sequenced.

### Data analysis

The obtained sequencing data from scRNAseq/snRNAseq were mapped to a genome of the *D. melanogaster* strain Oregon-R (OreR) (FBsn0000276) and reads mapped to individual genes were counted using 10 × Genomics Cellranger 5 using default settings for mapping single-cell data. For mapping single-nuclei data, the option –include-introns was added. The OreR genome was annotated by transferring the annotation of *D. melanogaster* genome r6.37 to a previously sequenced genome of Oregon-R [64, 118] using Liftoff [119].

Further data analyses were performed using R version 4.1.1 (2021-08-10). Specifically, the package Seurat [120] was used for single-cell specific applications. This includes quality control steps such as calculating the percentage of mitochondrial, ribosomal and heat shock related genes and removing doublets and cells or nuclei of poor quality. Cells of poor quality were defined as expressing more than 3000 or less than 300 genes. Nuclei of poor quality were defined as the top 1% of nuclei expressing the highest number of genes or less than 300 genes. As a threshold of 5–10% of mitochondrial reads per cell resulted in a single cluster defined by mitochondrial genes (in scRNAseq, none in snRNAseq), we decided to exclude cells and nuclei with more than 10% of mitochondrial reads to include as much data as possible for further analyses. Genes were kept if they were expressed in at least 5 cells (for scRNAseq) or 3 nuclei (for snRNAseq). Normalization was performed using the SCTransform method [121]. Unbiased clustering was performed in Seurat [122] and marker genes enriched in each cell cluster were identified by differential expression analyses (i.e. genes expressed in a cluster *vs.* all other clusters) followed by a cutoff of log2-fold change > 0.25 and an adjusted p-value < 0.05 (list of cell cluster markers for scRNAseq: Additional file 4: Table S4; list of cell cluster markers for snRANseq: Additional file 6: Table S6). Marker genes for each cell cluster were used to test for enrichment of gene ontology (GO) terms (i.e. Biological Process) using the R package gprofiler2 [123, 124] (GO enrichment for scRNAseq: Additional file 5: Table S5; GO enrichment for snRNAseq: Additional file 7: Table S7). The top four genes defining each cell cluster were chosen by the lowest adjusted *p*-value.

To compare the most variable genes between scRNAseq and snRNAseq, the top 3000 variable genes for both datasets were obtained based on the differential expression analysis (see above). Both lists were compared to identify those genes that were unique for each dataset and those that were shared. A gene ontology enrichment analysis was performed for the two lists of unique genes and the list of shared genes, respectively, using Gene Ontology. Gene ontology enrichment plots were created using ShinyGO (version 0.76) [125]. Radar plots were generated using Excel.

For the analysis of integrated data from both single cells and single nuclei, we applied the standard workflow in Seurat [126]. We used 3000 integration features and used FindIntegrationAnchors and IntegrateData adapted for datasets normalized using SCtransform. Barplots of dataset-specific celltype proportions were created using the R package dittoSeq [127]. The percentages of cells or nuclei in these plots were corrected for absolute number by multiplying the percentages in cells by 0.74, the ratio of filtered nuclei to filtered cells. UMAP plots were created using Seurat. Each dot represents a single cell or nucleus. They are positioned based on their relative transcriptional similarity to each other. Clusters were identified using a nearest neighbor clustering algorithm and the resolution for the depicted number of clusters was chosen based on visual inspection using the R package clustree [128]. Clusters were annotated by performing a differential gene expression analysis and scoring the overexpressed differentially expressed genes using a matrix of marker genes (Additional file 2: Table S2). To obtain comparable clusters across datasets, similar clusters were manually merged (Additional file 3: Table S3). Variable genes were obtained from the Seurat object and plotted using ggvenn (version 0.1.9.) [129]. Alluvial plots were created using the R package ggalluvial (version 0.12.3) [130].

The ggplot2 package was used to create plots if not otherwise stated. Note that all scripts and the entire analysis pipeline are available online (https://doi.org/10.25625/YHG4ET).

## Supplementary Information

**Additional file 1: Fig. S1.** FACS-plot of *D. melanogaster* eye-antennal disc cells after live/dead cell staining. (A) Counterstaining of Propidium Iodide to label dead cells (y-axis, Q1) and Calcein violet to label live cells (x-axis, Q4). Double positive signals might indicate dying cells or incompletely separated cells (Q2). This method allows removing debris (Q3) efficiently. (B) The cell population P4 (i.e. 16,208 living cells) were isolated and used for the scRNAseq run using 10x Genomics. **Fig. S2.** Quality and quantity of cDNA after reverse transcription of mRNA fraction (polyA-based enrichment) and full-length cDNA amplification from cell lysate of 30 cells sorted from cell suspension of eye-antennal discs run on Fragment Analyzer (Agilent). Size distribution of all fragments shows little impact on degradation (almost no cDNA detectable below 400 bp). **Fig. S3.** Contribution of mitochondrial gene expression to scRNAseq and snRNAseq datasets. (A) Total amount of genes (features) over percentage of mitochondrial reads, per cell each. The dashed line indicates a threshold of 10% of reads attributed to mitochondrial genes. In scRNAseq data, approximately 14% of cells show a high (>10%) proportion of mitochondrial gene reads on the total number of reads. (B) Total amount of genes (features) over Percentage of mitochondrial reads, per cell each in snRNAseq data. The dashed line indicates a threshold of 10% of reads attributed to mitochondrial genes. In most nuclei, only a low percentage of reads is attributed to mitochondrial genes. **Fig. S4.** Clustering and cluster annotation for scRNAseq data. (A) The heatmap shows the score for each potential cell type (Y-axis) in each cluster (X-axis). The cell types are annotated based on the highest scoring identity in the heatmap. The clusters are grouped based on their transcriptional similarity to each other. For clusters which express an equal number of marker genes for two different identities both identities were assigned (e.g. cluster 3:Antenna|EAB). Clusters with unresolved identities (i.e. more than two equal assignments) are called “Other”. The colors of the cluster names correspond to the colors in UMAP in (B). The marker score is calculated using a matrix of published marker genes (see Additional file Table S2). (B) UMAP of scRNAseq data. The clusters were annotated based on the heatmap in (A). This UMAP is identical to the UMAP with combined cluster annotation shown in Fig. 3A. Note that the color code is not comparable to the one used in Additional file Figs. S7 and S10. **Fig. S5.** Fluorescence intensity curves from Bioanalyzer for fresh- and cryopreserved nuclei obtained by different nuclei extraction protocols. (A) The RNA was extracted directly from a fresh sample (36 eye-antennal discs), which was dissociated using the 10x Genomics protocol with 0.1% IGEPAL as a detergent. (B) RNA isolated from a cryopreserved sample, which was dissociated using a protocol based on Triton X-100 as a detergent and a variety of RNAse inhibitors [1]. Note that the sample was thawed for 3.5h before being frozen again. Both curves are close to the expectation of RNA isolated from *D. melanogaster* [2]. **Fig. S6.** BioAnalyzer results comparing different nuclei isolation protocols for frozen samples. Samples 1, 2, 4 and 5 were dissociated using the protocol based on Triton X-100 as a detergent and a variety of RNAse inhibitors [1]. Samples 1 and 2 were dissociated by pipetting up and down and samples 4 and 5 were dissociated using a Dounce homogenizer. Samples 7 and 8 were dissociated using the protocol “10x Genomics^®^ Isolation of Nuclei for Single Cell RNA Sequencing” [3]. Samples 7 and 8 were dissociated using only citric acid buffer and samples 10 und 11 were dissociated using only a detergent. Note that for *D. melanogaster*, intense bands are expected at about 40s and weaker bands at 25s and 45-50s [2]. Each run was repeated once. **Fig. S7.** Clustering and cluster annotation for snRNAseq data. (A) The heatmap shows the score for each potential cell type (Y-axis) in each cluster (X-axis). The cell types are annotated based on the highest scoring identity in the heatmap. The clusters are grouped based on their transcriptional similarity to each other. For clusters which express an equal number of marker genes for two different identities both identities were assigned (e.g. cluster 15:MFurrow|SMW). Clusters with unresolved identities (i.e. more than two equal assignments) are called “Other”. The colors of the cluster names correspond to the colors in UMAP in (B). The marker score is calculated using a matrix of published marker genes (see Additional file Table S2). (B) UMAP of snRNAseq data. The clusters were annotated based on the heatmap in (A). This UMAP is identical to the UMAP with combined cluster annotation shown in Fig. 3C. Note that the color code is not comparable to the one used in Additional file Figs. S4 and S10. **Fig. S8.** Gene ontology enrichment analysis for genes with most variable expression. (A) Top 3000 genes unique to scRNAseq (i.e. 1520 genes). (B) Top 3000 genes unique to snRNAseq (i.e. 1520 genes). (C) Top 3000 genes shared between scRNAseq and snRNAseq (i.e. 1480 genes). See also Supplementary Table S9 for a full list of enriched GO terms. **Fig. S9.** Comparison of dataset specific and shared differentially expressed genes for each cell type. The radar plot shows for each cell type the percentage of cluster specific differentially expressed genes unique for the scRNAseq and snRNAseq data, respectively (red and blue lines), as well as the percentage of differentially expressed genes shared between both datasets (black line). The total number of genes fulfilling the differential expression criteria (FDR 0.05 and log2-fold change > 0.25) for each cell type is shown in brackets. **Fig. S10.** Clustering and cluster annotation of integrated scRNAseq and snRNAseq dataset. (A) The heatmap shows the score for each potential cell type (Y-axis) in each cluster (X-axis). The cell types are annotated based on the highest scoring identity in the heatmap. The clusters are grouped based on their transcriptional similarity to each other. For clusters which express an equal number of marker genes for two different identities both identities were assigned (e.g. cluster 14:Dorsal|Ocelli). Clusters with unresolved identities (i.e. more than two equal assignments) are called “Other”. The colors of the cluster names correspond to the colors in the UMAP in (B). The marker score is calculated using a matrix of published marker genes (see Additional file Table S2). (B) UMAP of integrated scRNAseq and snRNAseq data. Cells are colored by clusters identified based on the (A). Note that the color code in A and B is not comparable to the one used in Additional file Figs. S4 and S7. **Table S1.** Overview of different dissociation conditions. Samples within blocks (highlighted in grey and white) were prepared in parallel. The Flow Cytometer only provides percentages of survival because it stops after a defined number of events (i.e. ~50,000 cells) and therefore absolute numbers are not meaningful. “Pipetting” refers to the number of strokes during and after incubation. The cells obtained by experiment/block 12 were subjected to 10X Genomics scRNAseq.

**Additional file 2: Table S2.** Score Matrix used to annotate cell types and list of references for individual marker genes used for cluster/cell type annotation.

**Additional file 3: Table S3.** Results of automatic cluster annotation and assignment to combined clusters.

**Additional file 4: Table S4.** List of marker genes for each cluster in scRNAseq analysis.

**Additional file 5: Table S5.** GO analysis of cell clusters identified in scRNAseq.

**Additional file 6: Table S6.** List of marker genes for each cluster in snRNAseq analysis.

**Additional file 7: Table S7.** GO analysis of cell clusters identified in snRNAseq.

**Additional file 8: Table S8.** List of top 3000 variable genes for scRNAseq and snRNAseq data, respectively.

**Additional file 9: Table S9.** Top 3000 variable genes and GO enrichment results for scRNAseq, snRNAseq and shared.

**Additional file 10: Table S10.** Comparison of differentially expressed genes for each comparable cluster between scRNAseq and snRNAseq data.

## Data Availability

All Additional files tables and figures are part of this submission. All scripts and the entire analysis pipeline are available online (https://doi.org/10.25625/YHG4ET).
