# Supplementary material for: Tissue dissociation for single-cell and single-nuclei RNA sequencing for low amounts of input material
Source: Front Zool. 2022 Nov 12;19:27. doi: 10.1186/s12983-022-00472-x (PMC9652833; doi:10.1186/s12983-022-00472-x)
Supplement: Supplementary file 1 — Additional file 1: Fig. S1. FACS-plot of D. melanogaster eye-antennal disc cells after live/dead cell staining. (A) Counterstaining of Propidium Iodide to label dead cells (y-axis, Q1) and Calcein violet to label live cells (x-axis, Q4). Double positive signals might indicate dying cells or incompletely separated cells (Q2). This method allows removing debris (Q3) efficiently. (B) The cell population P4 (i.e. 16,208 living cells) were isolated and used for the scRNAseq run using 10x Genomics. Fig. S2. Quality and quantity of cDNA after reverse transcription of mRNA fraction (polyA-based enrichment) and full-length cDNA amplification from cell lysate of 30 cells sorted from cell suspension of eye-antennal discs run on Fragment Analyzer (Agilent). Size distribution of all fragments shows little impact on degradation (almost no cDNA detectable below 400 bp). Fig. S3. Contribution of mitochondrial gene expression to scRNAseq and snRNAseq datasets. (A) Total amount of genes (features) over percentage of mitochondrial reads, per cell each. The dashed line indicates a threshold of 10% of reads attributed to mitochondrial genes. In scRNAseq data, approximately 14% of cells show a high (>10%) proportion of mitochondrial gene reads on the total number of reads. (B) Total amount of genes (features) over Percentage of mitochondrial reads, per cell each in snRNAseq data. The dashed line indicates a threshold of 10% of reads attributed to mitochondrial genes. In most nuclei, only a low percentage of reads is attributed to mitochondrial genes. Fig. S4. Clustering and cluster annotation for scRNAseq data. (A) The heatmap shows the score for each potential cell type (Y-axis) in each cluster (X-axis). The cell types are annotated based on the highest scoring identity in the heatmap. The clusters are grouped based on their transcriptional similarity to each other. For clusters which express an equal number of marker genes for two different identities both identities were assigned (e.g [file 12983_2022_472_MOESM1_ESM.docx]

Supplementary Material

**Tissue dissociation for single-cell and single-nuclei RNA-sequencing for low amounts of input material**

Gordon Wiegleb^1,2^, Susanne Reinhardt^3^, Andreas Dahl^3^, Nico Posnien^1,4^ *

*correspondence: [nposnie@gwdg.de](https://gwdg-my.sharepoint.com/personal/nposnie_gwdg_de/Documents/Daten/Postdoc/Paper_Writing/scRNAseq_Methods_Paper/manuscript_snRNA_method/nposnie@gwdg.de)

**This PDF file contains:**

Supplementary Table S1

Supplementary Figures S1-S10

**The following Supplementary Tables are available separately as Excel files:**

Supplementary Table S2: Score Matrix used to annotate cell types and list of references for individual marker genes used for cluster/cell type annotation.

Supplementary Table S3: Results of automatic cluster annotation and assignment to combined clusters.

Supplementary Table S4: List of marker genes for each cluster in scRNAseq analysis.

Supplementary Table S5: GO analysis of cell clusters identified in scRNAseq.

Supplementary Table S6: List of marker genes for each cluster in snRNAseq analysis.

Supplementary Table S7: GO analysis of cell clusters identified in snRNAseq.

Supplementary Table S8: List of top 3,000 variable genes for scRNAseq and snRNAseq data, respectively.

Supplementary Table S9: Top 3,000 variable genes and GO enrichment results for scRNAseq, snRNAseq and shared.

Supplementary Table S10: Comparison of differentially expressed genes for each comparable cluster between scRNAseq and snRNAseq data.

**Scripts used for data analyses:**

Online repository: <https://doi.org/10.25625/YHG4ET>

**Supplementary Table S1. Overview of different dissociation conditions.**

Samples within blocks (highlighted in grey and white) were prepared in parallel. The Flow Cytometer only provides percentages of survival because it stops after a defined number of events (i.e. ~50,000 cells) and therefore absolute numbers are not meaningful. “Pipetting” refers to the number of strokes during and after incubation. The cells obtained by experiment/block 12 were subjected to 10X Genomics scRNAseq.


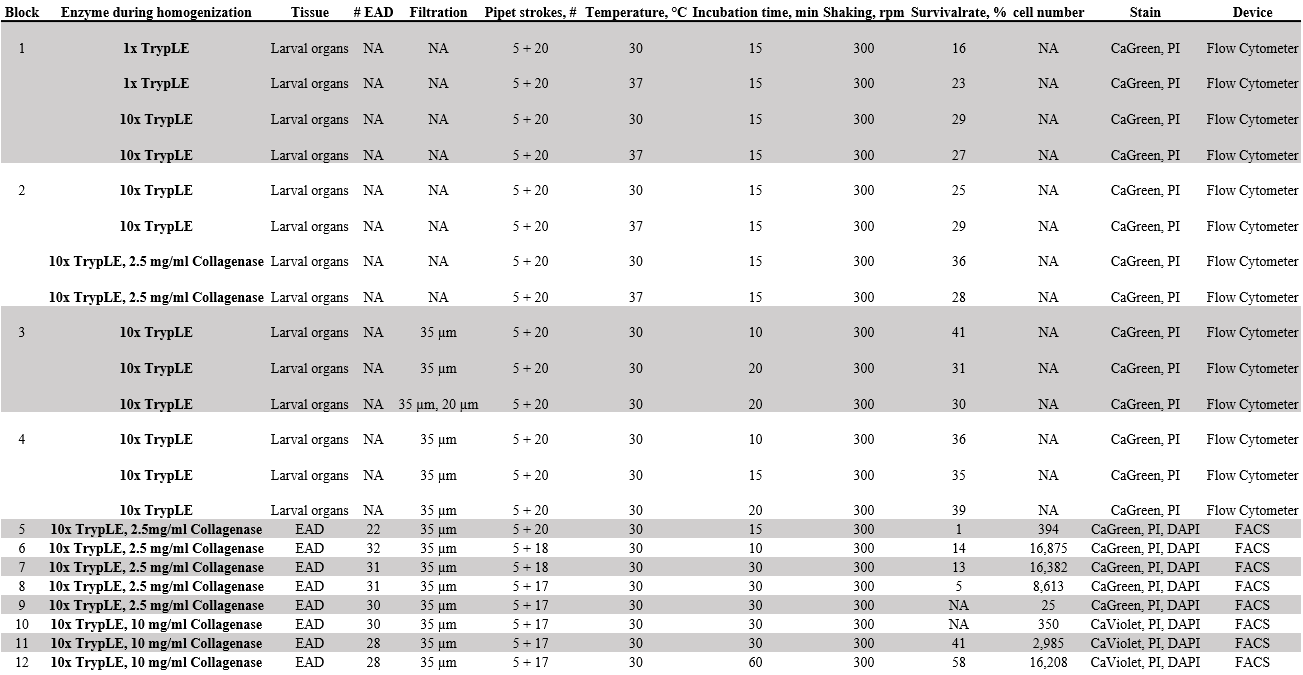


| **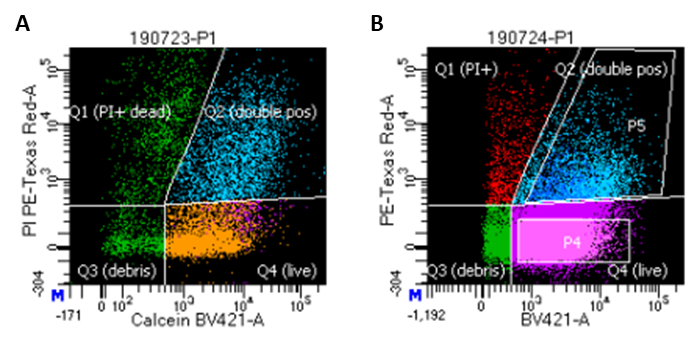** |
| --- |

**Supplementary Figure S1.** **FACS-plot of *D. melanogaster* eye-antennal disc cells after live/dead cell staining.**

(**A**) Counterstaining of Propidium Iodide to label dead cells (y-axis, Q1) and Calcein violet to label live cells (x-axis, Q4). Double positive signals might indicate dying cells or incompletely separated cells (Q2). This method allows removing debris (Q3) efficiently. (**B**) The cell population P4 (i.e. 16,208 living cells) were isolated and used for the scRNAseq run using 10x Genomics.

| 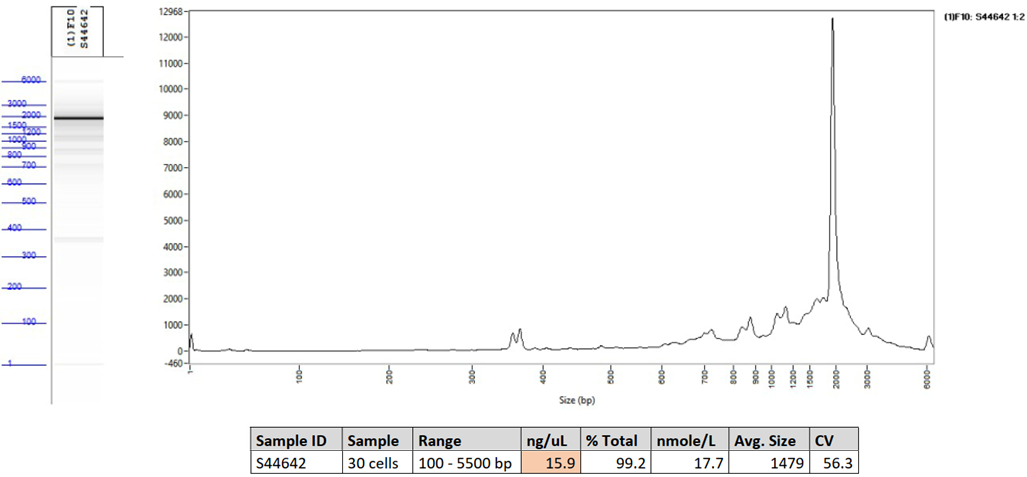 |
| --- |

**Supplementary Figure S2.** **Quality and quantity of cDNA after reverse transcription of mRNA fraction (polyA-based enrichment) and full-length cDNA amplification from cell lysate of 30 cells sorted from cell suspension of eye-antennal discs run on Fragment Analyzer (Agilent)**.

Size distribution of all fragments shows little impact on degradation (almost no cDNA detectable below 400 bp).

| 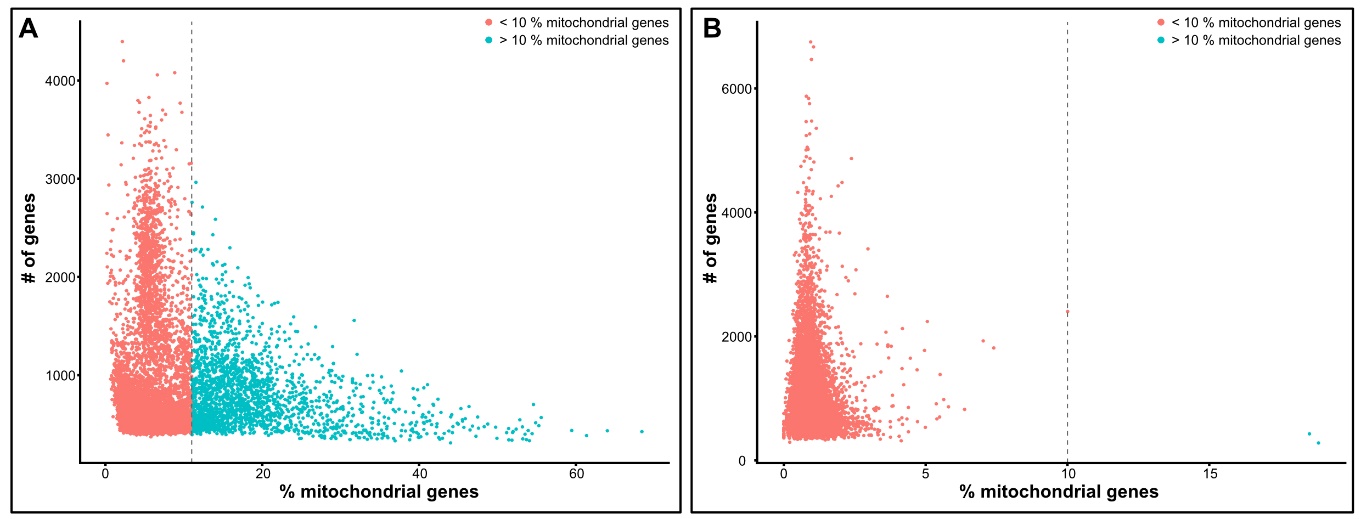 |
| --- |

**Supplementary Figure S3.** **Contribution of mitochondrial gene expression to scRNAseq and snRNAseq datasets**.

(**A**) Total amount of genes (features) over percentage of mitochondrial reads, per cell each. The dashed line indicates a threshold of 10% of reads attributed to mitochondrial genes. In scRNAseq data, approximately 14% of cells show a high (>10%) proportion of mitochondrial gene reads on the total number of reads. (**B**) Total amount of genes (features) over Percentage of mitochondrial reads, per cell each in snRNAseq data. The dashed line indicates a threshold of 10% of reads attributed to mitochondrial genes. In most nuclei, only a low percentage of reads is attributed to mitochondrial genes.


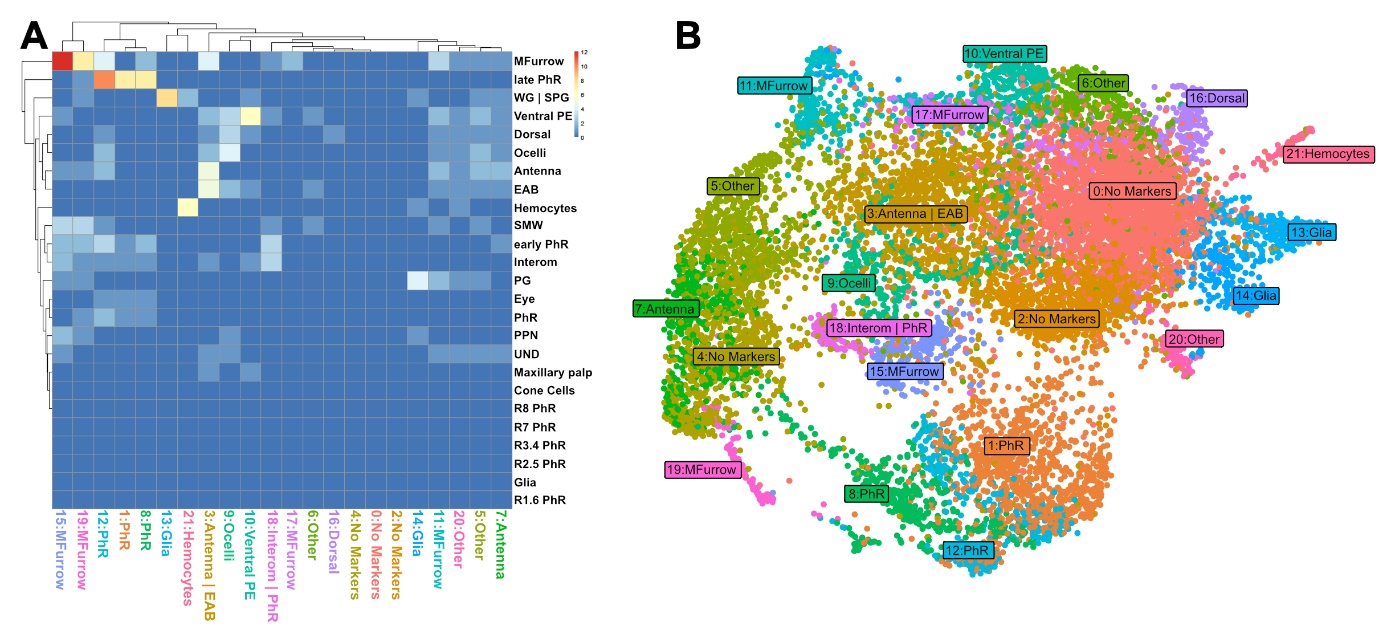


Supplementary Figure S4. Clustering and cluster annotation for scRNAseq data.

**(A)** The heatmap shows the score for each potential cell type (Y-axis) in each cluster (X-axis). The cell types are annotated based on the highest scoring identity in the heatmap. The clusters are grouped based on their transcriptional similarity to each other. For clusters which express an equal number of marker genes for two different identities both identities were assigned (e.g. cluster 3:Antenna | EAB). Clusters with unresolved identities (i.e. more than two equal assignments) are called “Other”. The colors of the cluster names correspond to the colors in UMAP in **(B)**. The marker score is calculated using a matrix of published marker genes (see Supplementary Table S2). **(B)** UMAP of scRNAseq data. The clusters were annotated based on the heatmap in **(A)**. This UMAP is identical to the UMAP with combined cluster annotation shown in Figure 3A. Note that the color code is not comparable to the one used in Supplementary Figures S7 and S10.

| 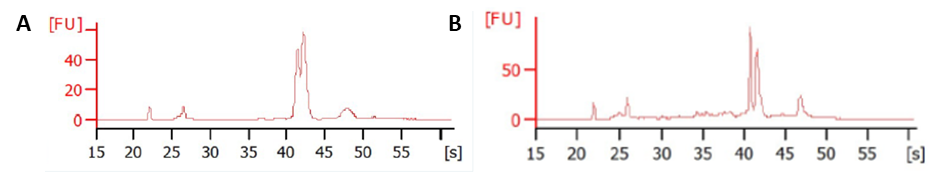 |
| --- |

**Supplementary Figure S5. Fluorescence intensity curves from Bioanalyzer for fresh- and cryopreserved nuclei obtained by different nuclei extraction protocols.**

(**A**) The RNA was extracted directly from a fresh sample (36 eye-antennal discs), which was dissociated using the 10x Genomics protocol with 0.1% IGEPAL as a detergent. (**B**) RNA isolated from a cryopreserved sample, which was dissociated using a protocol based on Triton X-100 as a detergent and a variety of RNAse inhibitors [1]. Note that the sample was thawed for 3.5h before being frozen again. Both curves are close to the expectation of RNA isolated from *D. melanogaster* [2].

| 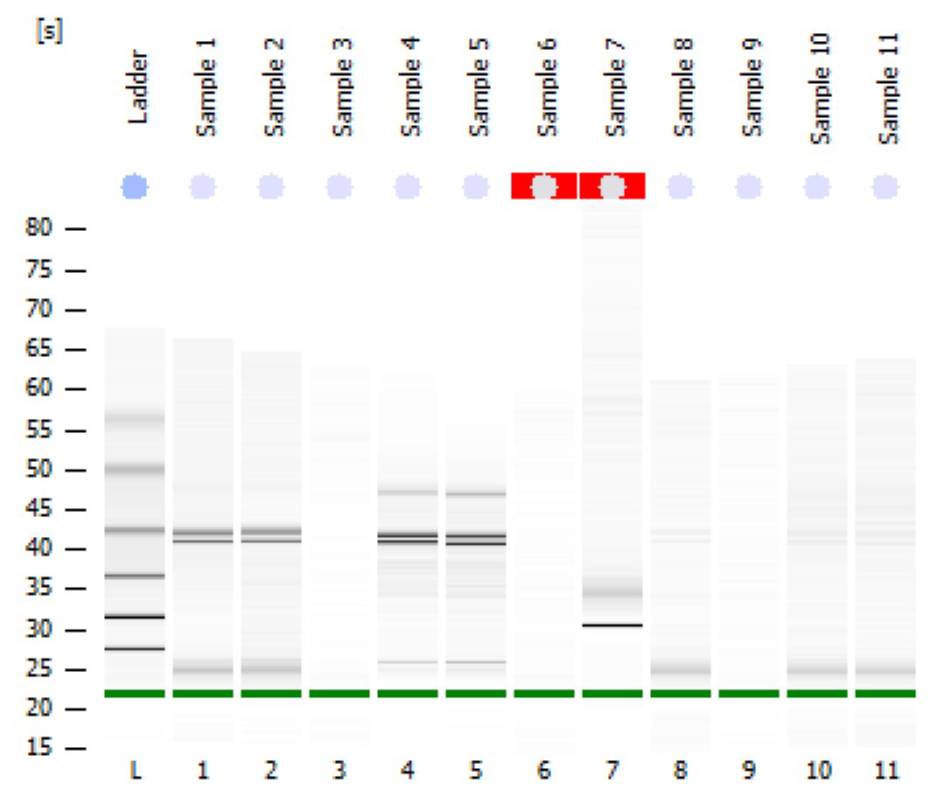 |
| --- |

Supplementary Figure S6. BioAnalyzer results comparing different nuclei isolation protocols for frozen samples.

Samples 1, 2, 4 and 5 were dissociated using the protocol based on Triton X-100 as a detergent and a variety of RNAse inhibitors [1]. Samples 1 and 2 were dissociated by pipetting up and down and samples 4 and 5 were dissociated using a Dounce homogenizer. Samples 7 and 8 were dissociated using the protocol “10x Genomics® Isolation of Nuclei for Single Cell RNA Sequencing” [3]. Samples 7 and 8 were dissociated using only citric acid buffer and samples 10 und 11 were dissociated using only a detergent. Note that for *D. melanogaster*, intense bands are expected at about 40s and weaker bands at 25s and 45-50s [2]. Each run was repeated once.


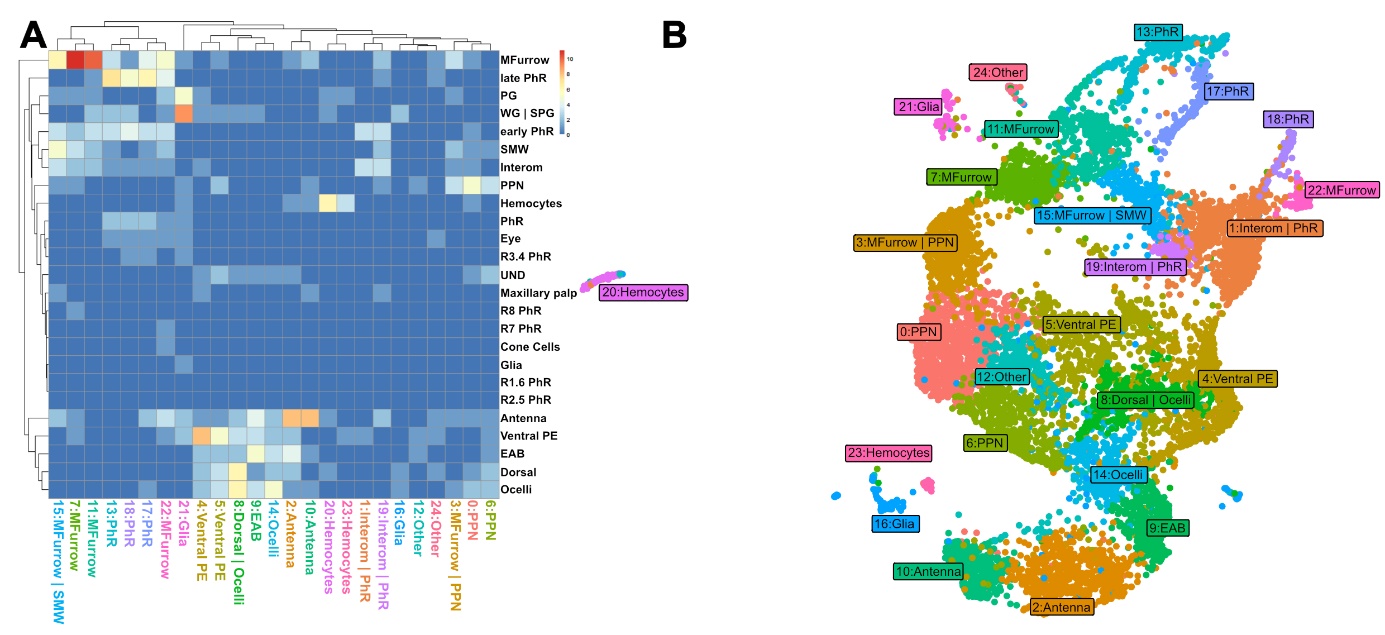


Supplementary Figure S7. Clustering and cluster annotation for snRNAseq data.

(A) The heatmap shows the score for each potential cell type (Y-axis) in each cluster (X-axis). The cell types are annotated based on the highest scoring identity in the heatmap. The clusters are grouped based on their transcriptional similarity to each other. For clusters which express an equal number of marker genes for two different identities both identities were assigned (e.g. cluster 15:MFurrow | SMW). Clusters with unresolved identities (i.e. more than two equal assignments) are called “Other”. The colors of the cluster names correspond to the colors in UMAP in (B). The marker score is calculated using a matrix of published marker genes (see Supplementary Table S2). (B) UMAP of snRNAseq data. The clusters were annotated based on the heatmap in (A). This UMAP is identical to the UMAP with combined cluster annotation shown in Figure 3C. Note that the color code is not comparable to the one used in Supplementary Figures S4 and S10.

| 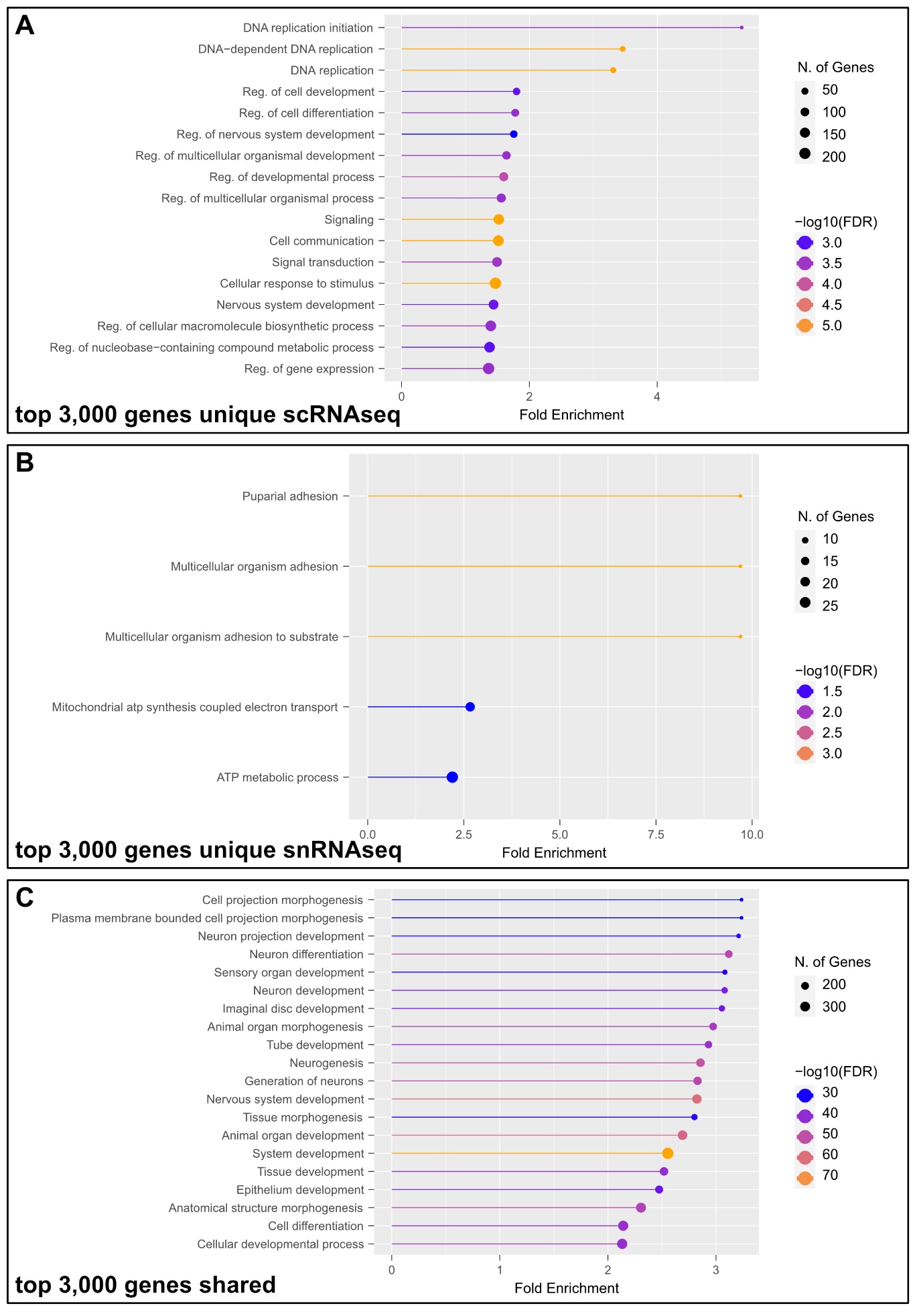 |
| --- |

Supplementary Figure S8. Gene ontology enrichment analysis for genes with most variable expression.

(**A**) Top 3,000 genes unique to scRNAseq (i.e. 1,520 genes). (**B**) Top 3,000 genes unique to snRNAseq (i.e. 1,520 genes). (**C**) Top 3,000 genes shared between scRNAseq and snRNAseq (i.e. 1,480 genes). See also Supplementary Table S9 for a full list of enriched GO terms.


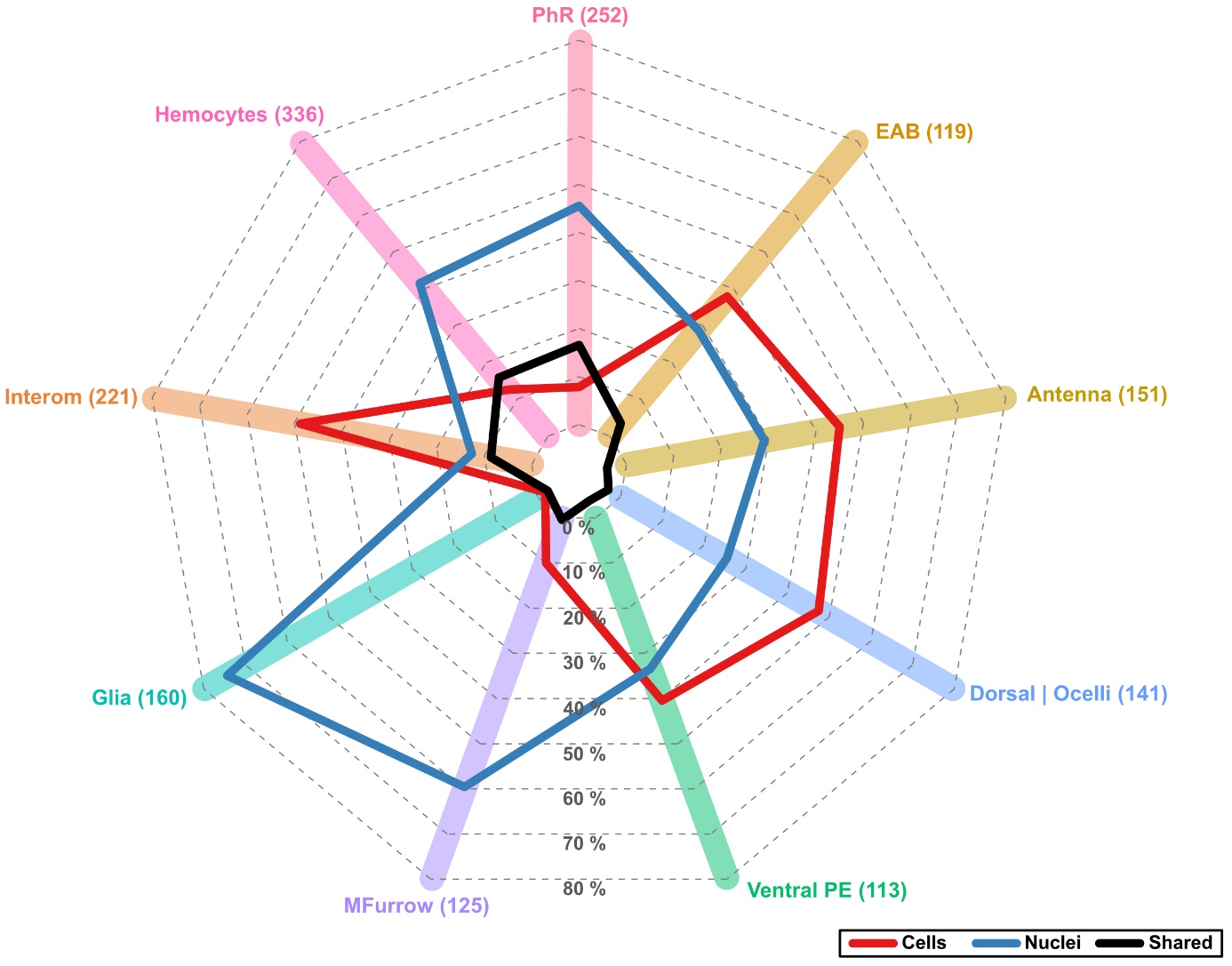


Supplementary Figure S9. Comparison of dataset specific and shared differentially expressed genes for each cell type.

The radar plot shows for each cell type the percentage of cluster specific differentially expressed genes unique for the scRNAseq and snRNAseq data, respectively (red and blue lines), as well as the percentage of differentially expressed genes shared between both datasets (black line). The total number of genes fulfilling the differential expression criteria (FDR 0.05 and log2-fold change > 0.25) for each cell type is shown in brackets.


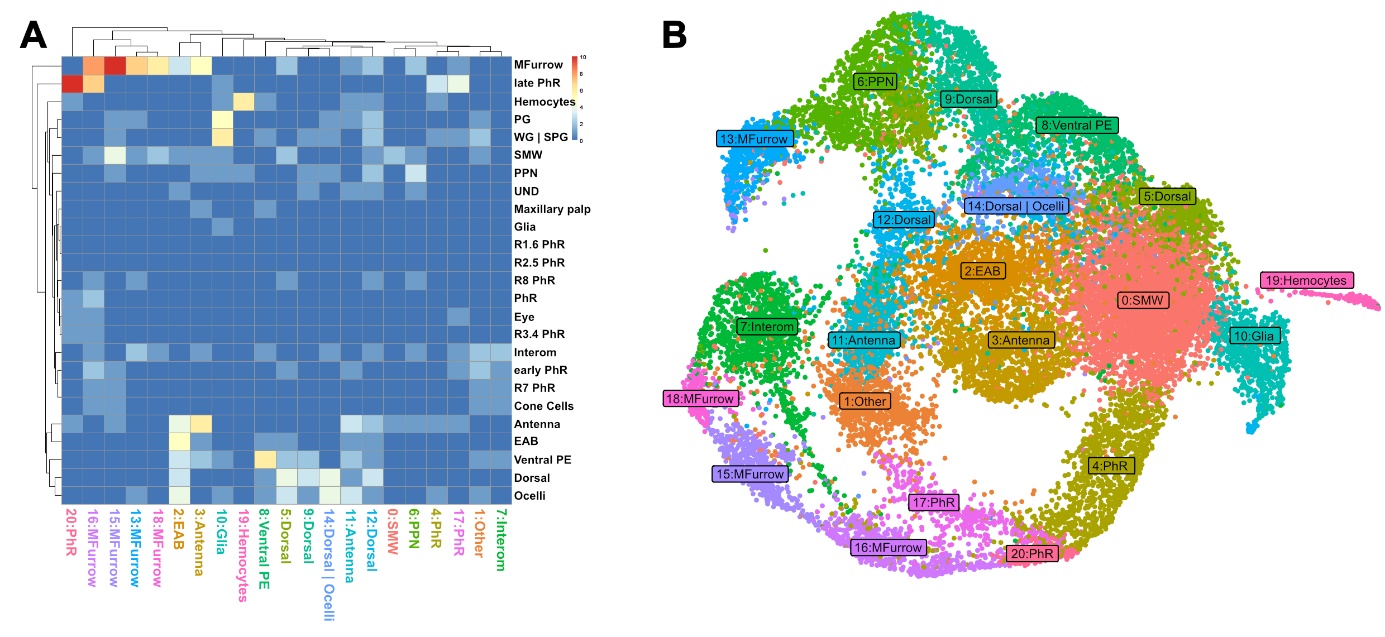


Supplementary Figure S10. Clustering and cluster annotation of integrated scRNAseq and snRNAseq dataset.

**(A)** The heatmap shows the score for each potential cell type (Y-axis) in each cluster (X-axis). The cell types are annotated based on the highest scoring identity in the heatmap. The clusters are grouped based on their transcriptional similarity to each other. For clusters which express an equal number of marker genes for two different identities both identities were assigned (e.g. cluster 14:Dorsal | Ocelli). Clusters with unresolved identities (i.e. more than two equal assignments) are called “Other”. The colors of the cluster names correspond to the colors in the UMAP in **(B)**. The marker score is calculated using a matrix of published marker genes (see Supplementary Table S2). **(B)** UMAP of integrated scRNAseq and snRNAseq data. Cells are colored by clusters identified based on the **(A)**. Note that the color code in A and B is not comparable to the one used in Supplementary Figures S4 and S7.

References

1. Litvinukova M, Lindberg E, Maatz H, Zhang H, Radke M, Gotthardt M, et al. Single Cell and Single Nuclei Analysis Human Heart Tissue. protocols.io 2018. doi:10.17504/protocols.io.veae3ae.

2. Jeffrey A. Fabrick JA, Hull JJ. Assessing Integrity of Insect RNA: Application Note. 2017. https://www.ars.usda.gov/ARSUserFiles/20200500/Pubs%202018/FabrickHull%20AgilentNot4es2017.pdf. Accessed 28 Apr 2022.

3. 10X Genomics. Nuclei Isolation from Cell Suspensions & Tissues for Single Cell RNA Sequencing: CG000124 • Rev F. 2021. https://assets.ctfassets.net/an68im79xiti/2HNFgXau0ntv1BhS4ffn6n/71a29daf18e5f7c30cf06b5b4f829e44/CG000124_Demonstrated_Protocol_Nuclei_isolation_RevF.pdf. Accessed 28 Apr 2022.
